# Age-related decline of various cognitive functions in well-experienced male rats treated with the putative anti-aging compound (2*R*)-1-(1-benzofuran-2-yl)-N-propylpentane-2-amine ((-)BPAP)

**DOI:** 10.1007/s11357-023-00821-6

**Published:** 2023-06-12

**Authors:** Aliz Judit Ernyey, Ferenc Kassai, Kata Kozma, Imola Plangár, Zsuzsa Somfai, Ildikó Miklya, István Gyertyán

**Affiliations:** 1 MTA-SE NAP B Cognitive Translational Behavioural Pharmacology Group, Department of Pharmacology and Pharmacotherapy, Faculty of Medicine, Semmelweis University, Nagyvárad Tér 4, H-1089 Budapest, Hungary; 2 Department of Pharmacology and Pharmacotherapy, Faculty of Medicine, Semmelweis University, Nagyvárad Tér 4, 1089 Budapest, Hungary

**Keywords:** Age-related changes, Cognitive enhancer, Learning, Memory, Enhancer regulation

## Abstract

Aging-associated cognitive disorders lack proper medication. To meet this need translation-wise, modification of the animal models is also required. In the present study, effect of the putative anti-aging compound (2*R*)-1-(1-benzofuran-2-yl)-N-propylpentane-2-amine ((-)BPAP, a deprenyl derivative) on age-related cognitive decline was investigated in experienced, aged Long-Evans rats. During their lifetime, animals had acquired knowledge in various cognitive assays. Their performance in these tests was then parallel followed from the age of 27 months until their death meanwhile half of them were treated with BPAP. Cognitive performance in various tasks showed different sensitivities/resistances to age-related impairment. Pot jumping performance (motor skill-learning) started to impair first, at 21 months of age, followed by decreasing performance in five-choice serial reaction time task (attention) at 26 months. Navigation performance in Morris water maze (spatial learning) started to decline at 31 months. Performance in a cooperation task (social cognition) started to decline the latest, at 34 months. Our findings suggest that in this process, the primary factor was the level of motivation to be engaged with the task and not losing the acquired knowledge. The average lifespan of the tested rat population was 36 months. BPAP could not improve the cognitive performance; neither could it prolong lifespan. A possible reason might be that dietary restriction and lifelong cognitive engagement had beneficial effects on cognitive capabilities and lifespan creating a “ceiling effect” for further improvement. The results confirmed that experienced animals provide a translationally relevant model to study age-related cognitive decline and measure the effect of putative anti-aging compounds.

**Supplementary Information:**

The online version contains supplementary material available at 10.1007/s11357-023-00821-6.

## Introduction

The otherwise welcome prolongation in life expectancy in modern society also brought along an increased frequency of age-associated diseases, such as various types of dementia. Loss of cognitive abilities not only inflict the life of the patients but also put high burden on their close relative caregivers and the whole health care system. The financial costs are estimated to exceed and exhaust the socially affordable capacities by 2050, unless these diseases can be effectively medicated. Novel compounds that have cognitive enhancer effects and/or can modify the disease progress are of great significance in this race with time. Unfortunately, development of new cognitive enhancer drugs has been anything but a success story in the past two decades [1–3].

The reasons behind the serial failures of novel drug candidates are manifold, but the low predictive power of the applied animal models is certainly a major one. Regarding aging models, the relative short duration of the studies using either accelerated aging models or species of much shorter lifespan than that of man constitutes a major translational problem [4–6], while in area of age-related diseases, the not enough prudent reliance on hypothesized disease pathomechanism (see the case of amyloid theory in Alzheimer’s disease) and the overwhelming and uncritical use of transgenic mouse models are mostly blamed for the failures [1, 5, 7, 8]. The (over)simplified cognitive defect paradigms form a common flaw of both fields [1, 8, 9]. With the aim to overcome these problems, we established a complex rodent cognitive test system [1, 10-12], which includes learning paradigms representing different, clinically relevant cognitive domains. We teach several cognitive tasks to the same cohort of animals thereby creating a population with “widespread knowledge.” These animals are then transformed to a “patient” population by exposing them to a learning impairing method. Aging can be considered as a natural way of impairment and effects of putative cognitive enhancers can be investigated in this “patient population” on the decline of cognitive functions acquired during their lifetime.

In the animal literature, age-dependent cognitive decline is investigated either in cross-sectional or longitudinal studies. The former (which is the more common type) can detect the effect of age but not that of acquired experience. Longitudinal studies (especially when combined with cross-sectional design [13, 14] can — in principle — show the effect of experience as well. But even in longitudinal studies, animals are only tested on a few occasions at discrete time points separated by several months; this kind of experimental design does not allow the animals to practice their task(s). Moreover, the vast majority of the studies end at 24 months of age. Measuring several cognitive functions simultaneously is even rarer [15], especially in longitudinal studies (an example is [16]). Our study design is peculiar in that we continuously investigate the same animal cohort during the course of aging parallel in various cognitive assays until their death. This design allows to compare the onset and rate of age-dependent decline of various cognitive functions.

Our animals were over 2 years old at the beginning of the current study, and they had acquired and practiced different cognitive skills across their lifespan. They gained experience in five-choice serial reaction time task (5-CSRTT, [17]), Morris water maze (MWM, [18]), a cooperation task (COOP, [19]) carried out in pairs, and a skill-learning task, “pot-jumping” (PJT, [20]). We followed their cognitive performance during aging and tested the effect of a putative anti-aging compound (2*R*)-1-(1-benzofuran-2-yl)-N-propylpentane-2-amine ((-)BPAP, further in the paper: BPAP) [21] on this process until their spontaneous death or humane endpoint euthanasia.

BPAP is a member of the group of enhancer substances. Knoll described [22] that endogenous and exogenous enhancer substances, like trace amines and (-)deprenyl/selegiline, respectively, facilitate the impulse propagation mediated release of monoamines in the brain given new therapeutic possibilities for the neuronal regulation. Deprenyl, which was originally introduced as a selective MAO-B inhibitor [23], is a prominent representative of this class of compounds and currently is the only substance on the market showing an enhancer effect [24]. Deprenyl has been shown to prolong lifespan in several studies and in different mammalian species: rats [25–30], mice [31, 32], Syrian hamsters [33], beagle dogs [34], and even Drosophila melanogaster [35]. BPAP was developed as a follow-up compound of deprenyl, a selective synthetic enhancer substance without any MAO-B activity [21]. It was shown to exert neuroprotective [21, 36], anti-apoptotic [37], and neurotrophic factor synthesis upregulating effects [38], in vitro, and was found to be effective in a mouse model of Parkinson’s disease [39]. With BPAP, only one longevity study has been carried out so far, in which BPAP prolonged the lifespan of rats [27]. However, effects of the compound on several cognitive domains in aged experienced rats have not been yet examined.

Thus, the aim of this study was twofold: (1) to investigate the differential effect of aging on various cognitive functions within the same subjects and (2) to examine the cognitive enhancer, anti-aging or life-prolonging effect of BPAP.

## Materials and methods

### Subjects

Subjects of the study were 30 male 27-month-old Long Evans (LE) rats (Janvier, France). The animals had been trained in several cognitive tests from their age of 1 month, and they continued this activity until death or until they were physically not capable to participate in the given task anymore. Some of them needed to be euthanized because of impaired physical status or tumor that impeded them in movements and taking part in cognitive tasks. At the age of 24 months, the animals participated in a study for 7 weeks in that 16 rats were treated with a serotonin 5-HT6 receptor antagonist for 13 days and 14 rats got saline injections [10]. The present longevity study started after a 7-week long wash-out period.

Body weight of animals fell in the range of 355–486 g at the beginning of the study. Animals were housed in groups of three in 1500-cm^2^ polycarbonate cages with paper tube and wooden chewing bricks as enrichment tools and were regularly exposed to handling throughout the measurements. They were kept on reversed light-dark cycle (dark phase from 4:00 am to 4:00 pm) and restricted food access (commercial pellet rat feed R/M-Z + H produced by SSniff Spezialdiäten GmbH). The amount of food was 45 g for 3 cage-mates supplied at the end of the dark phase, at 3:00 pm. Water was available ad libitum. (These holding conditions had been applied since the arrival of the animals at the lab.) During the lifetime of the animals in case of within-cage aggression, the aggressive individual (altogether 7 rats) was isolated to a separate cage whilst allowing to maintain visual, auditory, and olfactory contacts with the previous cage-mates. In case of a death, the survived cage-mates remained in pair or alone as we did not risk combining them with rats from other cages.

The experiments were authorized by the regional animal health authority in Hungary (resolution number PEI/001/3572-4/2014) and conformed to the Hungarian welfare legislation and the EU 63/2010 Directive.

### Treatment of the animals

(2*R*)-1-(1-benzofuran-2-yl)-N-propylpentane-2-amine (BPAP) was supplied by Fujimoto Pharmaceutical Company (Osaka, Japan). Fifteen rats were treated with BPAP from their age of 27 months until spontaneous death or euthanasia; 15 rats were treated with saline. BPAP or saline was administered subcutaneously daily, except for Saturdays and Sundays, in the morning about 1 h before the scheduled cognitive task. The available pharmacokinetic data on BPAP [40] show that after a single, subcutaneously administered dose of 1 mg/kg drug level peaked at 30 min in the brain and ~ 90% of the dose was eliminated within 72 h via the urine and the stool (elimination half-life was 5.5–5.8 h). The initial dose of BPAP was 0.0002 mg/kg, which was used for 7 weeks, and afterwards, it was increased to 0.001 mg/kg because of lack of observed effects (see more detailed reasoning in “Discussion”). This dose was administered until the end of the study. The compound was dissolved in saline and subcutaneously injected in a volume of 1 ml/kg. The solution for the treatment was prepared weekly from a stock solution of 1 mg/ml stored at − 20 °C.

### Behavioral tests

#### 5-choice serial reaction time test

The operant chamber (TSE, Germany) was equipped with five nose-poke modules. Animals were trained to nose-poke into a randomly chosen hole marked for 1 s. In half of the animals, the “classical” 5CSRTT paradigm was applied, where turning *on* the stimulus light served as a signal (“light on” version). For the rest of the population, a novel, “reversed” 5CSRTT method was used. Here, all the nose-poke modules were illuminated, and turning *off* the stimulus light in one of the holes was the signal (“light off” version).

In both paradigms, correct responses were rewarded with a pellet (45 mg purified dustless precision pellets, Bio-Serv) delivered into the magazine. Nose-poke into the magazine initiated the next trial. The animal made an incorrect response if nose-poked into one of the non-signaled holes; a premature response if nose-poked into any of the holes during the 5 s long inter-trial interval; and an omission if it did not respond to the stimulus during its duration plus a 5-s-long hold period. Incorrect and premature responses as well as omissions were punished with a 5-s time-out period when the house light was turned off (“light on” version) or on (“light off” version). Duration of a daily test session was 20 min. Rats were trained for the 5-CSRTT at their 1.5–4 months’ age in stages with gradually increasing difficulty. Our results showed that the “off” version was a little bit more difficult than the “on” version: It took 2 days more to the animals to acquire the task, but afterwards, the two groups showed similar performance [40, 41] After this period, rats participated in regular maintenance training involving 1–2 sessions a week until the above mentioned 5-HT_6_ study (*see* above; [10]). Afterwards, rats again participated in regular maintenance training involving 1–2 sessions a week until the end of their lives. In these sessions, stimulus duration (SD) was randomly varied between 1 s, 0.5 s, and 0.25 s. However, this seemed to be a big challenge for the rats; they gave up working that led to several omissions and fewer rewards in BPAP-treated as well as in control group. For this reason, SD was again confined to 1 s at 34 months of age, without varying SD values. For the whole treatment period, only data obtained from 1 s SD trials were analyzed. The primary outcome parameter was the % successful trials. The “on” and the “off” group was pooled as no difference was observed in their performance.

#### Cooperation task in the Skinner box

The assay is described in details in Kozma et al. [19]. Two rats were placed in the same Skinner box (MedAssociates, USA). The opposite walls of the chamber were equipped with one nose-poke module and one magazine for each. In order to obtain food reward, two animals had to perform simultaneous nose-pokes after a stimulus light was turned on in both modules. The nose-pokes at the opposite sides were regarded as simultaneous if the delay between them did not exceed 1 s. Non-simultaneous responses or repeated nose-pokes to the same module were punished with 5 s timeout. Rats were trained for the task in stages with gradually decreasing intervals allowed for the “simultaneous” nose-pokes from 10 to 1 s. The training and maintenance testing of the animals including the study with a serotonin 5-HT_6_ receptor antagonist at 24 months of age is described in detail by Gyertyán et al. [10]. Afterwards, during a 7-week-long period until the beginning of the current study two sessions of cooperation test was performed. By the time the current study started, 28 cooperating rats forming 14 pairs participated in the task. The animals performed the task in triads of consecutive daily sessions, two triads/month. A triad consisted of 1 day of FR1 session, then the next day FR2, and the third day again an FR1 session. FR2 was a modified version of the original task (FR1) with increased task difficulty: The first simultaneous nose-poke did not result in a reward but only in an “acknowledging” tone stimulus, and right after, a further simultaneous nose-poke (within 1 s) was required to get the reward [4]. In the first two sessions, respectively 6 and 4 pairs were “mixed” BPAP-saline pairs (i.e. one rat was treated with BPAP, the other with vehicle) then these “mixed” pairs were changed to BPAP-BPAP and saline-saline pairs. From their age of 37 months, rats performed only FR1 sessions. In case of a death, the rat that remained alone was not doing the test until another rat died. If rats died in even number from one session to another, the missing animal was replaced forming a new BPAP-BPAP or saline-saline pair, if possible. In the last 6 sessions, there was one mixed pair among the pairs. The primary outcome parameter was the percentage of successful trials. In analysis of the results, only FR1, but not FR2, data were considered during the whole treatment period.

#### Morris water maze

The task of the animals was to find a hidden 10-cm-diameter platform in a 190-cm-diameter, 60-cm-deep circular tank filled with 39 cm water (23 ± 1 °C). The platform was 1 cm under the water surface, in the southeast quadrant, at about 40 cm distance from the side wall of the pool. On the wall of the experimental room, extra-maze cues were placed in order to facilitate the orientation during swimming. Animals were trained on four consecutive days in three daily training trials with 30-min inter-trial intervals. They were placed in the water at the north, east, south, or west edge of the pool in systemic rotation and were given 180 s to escape to the hidden target. They were allowed to remain on the platform for 30 s and afterwards were taken out, dried by a cloth, and returned to their cage. Movement of animals was recorded with Smart v3.0 video tracking system software (Panlab, Spain).

Animals were got acquainted with the MWM paradigm at the age of 8 months (*n* = 13), 9.5 months (*n* = 10), or 10.5 months (*n* = 9). At the age of 18–19 months, they all went through a modified version of the task designed to measure a kind of episodic memory [42]. At age of 24 months, the animals performed the task several times within 2 weeks in the abovementioned serotonin 5-HT6 receptor antagonist study [10]. Afterwards, they performed the task once with 4 daily trials until the beginning of the BPAP/saline treatment. In the course of the present longevity study, the task was repeated every 2–3 weeks with rotating the platform location between the four quadrants from session to session, until the end of their lives or until they were physically capable to swim. At the age of 33 months of the animals, due to weakening physical condition, the cutoff time was reduced from 180 to 90 s for all the animals. The animals that could not find the platform due to physical weakness or swimming difficulties were rescued from the water. In data analysis, these animals were given a 90-s “cutoff” escape latency time from their age of 33 months. At age of 37 months, we stopped testing the rats in this experiment due to their physical incapability.

The primary performance parameter was the escape latency. For the comparative analysis of the four tasks’ results, we transformed this value to “remaining time” — calculated as 90 s minus escape latency — so that the better performance is indicated by a higher value in MWM task as well. Then, daily average of “remaining time” values in the 4 trials was used as individual values in the statistical calculation.

#### Pot jumping test

The test served to measure procedural learning capabilities and was designed according to Ernyey et al. [20]. Briefly, the experiment was carried out in the MWM tank, where 12 flower pots (16 cm high and 10 cm wide at the bottom) were placed upside down forming a circle. Distance between the centers of the adjacent pots gradually increased from 18 to 46 cm in anti-clockwise direction. The tank was filled with 6-cm-deep cold water to restrain rats climbing off the pots. During a session, animals were placed onto the start pot, which was within the shortest distance from the next pot. For 3 min, they could freely move on the pots and their behavior was observed and recorded using Smart v3.0 video tracking software (Panlab, Spain). The longest inter-pot distance jumped over was the primary performance parameter. Pot jumping training of the animals started at 4.5 months of age with once a month session frequency. From 13 to 15 months, sessions were run biweekly. After a break in training, from the age of 19 months until 24 months, the animals were tested biweekly. From the study with serotonin 5-HT_6_ receptor antagonist (24 months of age), they were trained monthly in this experiment. The detailed results of these rats’ lifelong performance in pot jumping task were already published [20].

### Statistical analysis

For statistical analysis, Statistica 13.5.0.17 software package (TIBCO Software Inc.) was used. For comparing the performance of the two treatment groups, first monthly mean of each rat’s output values was calculated, and then, group means of these means were calculated. Due to the monthly changing sample size, repeated measures ANOVA was not suitable for the statistical evaluation of the time course. We refrained from replacing data of missing animals by the “last observation carried forward” method, because it would have distorted the group means and we also rejected using the actual group mean as a replacing value as this procedure would have led to biased standard deviation. Therefore, monthly performance in the two treatment groups was compared with *t*-test for independent samples involving “observed cases” in the different cognitive tasks. Because of using multiple *t*-tests significance levels were adjusted by Holm-Bonferroni correction.

Survival data of the two groups were plotted by Kaplan-Meier method, and Cox-Mantel test was used for significance analysis.

To compare the effect of aging among the four cognitive domains, data of the two treatment groups were pooled. For each animal, a 3-month moving average was calculated in each task (average of month 1, month 2, and month 3 is shown as data of month 3). Afterwards, data were normalized by range. The range was the minimum and maximum performance in the whole population during their entire lifetime. The difference between the individual values and minimum values was divided by the range. Only observed cases were considered; therefore, monthly data were separately analyzed by univariate ANOVA (with “tests” as the grouping factor) and Duncan test was applied for post hoc comparisons. In this analysis, performance data of the animals from their age of 20 months were included.

### Autopsy

After death of the animals, the corpses of 25 rats (12 of them treated by BPAP and 13 rats treated by saline) were kept frozen until autopsy. Macroscopic examination was performed on the corpses. Two rats were still alive on the day of the autopsy (1 treated with BPAP and the other with saline), and 2 corpses were not available (both treated with BPAP), so these were not dissected. For autopsy, the corpses were thawed and the weight of the following organs was measured beside their macroscopic examination: the brain, lung, heart, liver, spleen, kidneys, adrenal, testicles and tumors, if found.

## Results

**Fig. 1:**
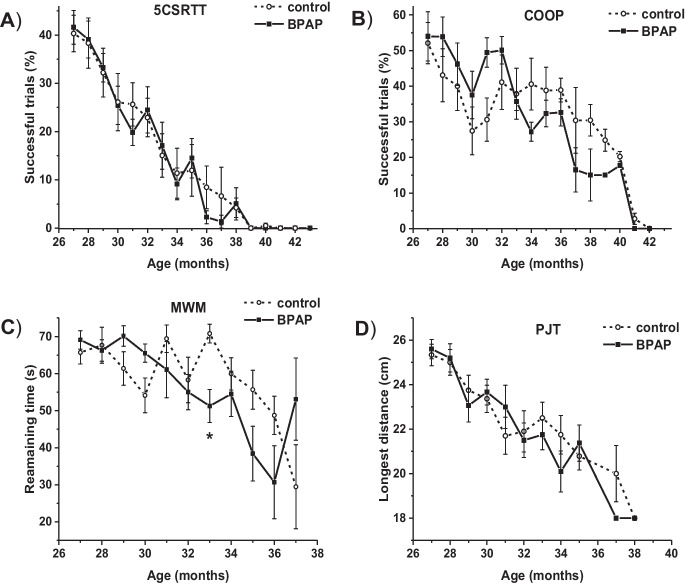
Performance of rats depending on their age from the beginning of the treatment with BPAP/saline (age of 27 months) in A) the 5-choice serial reaction time task (5CSRTT), B) cooperation task (COOP), C) Morris water maze experiment (MWM), D) „pot jumping” test (PJT). Solid line and filled square symbols represent the performance of BPAP-treated rats and dashed line with hollow circle symbols represent the performance of the control group. Mean ± s.e.m values are shown. *: p < 0.05 significant difference between the two groups (t-test for independent samples with Holm-Bonferroni correction)

As a first step, the performance of BPAP- and vehicle-treated groups were compared in each of the behavioral tasks (Fig. 1A: 5CSRTT, Fig. 1B: Coop, Fig. 1C: MWM, Fig. 1D: PJT). The number of rats participating in the tasks is listed in Table 1. In all the applied tasks, the difference was not significant between BPAP-treated and vehicle-treated groups during the whole measurement period. There was only one significantly different data point in MWM performance, at 33 months of age (Fig. 1C). The detailed statistical results are shown in Table S1, S2, S3, and S4 in *Supplementary material*.

**Table 1:** Number of rats participating in the applied tasks at different ages during the lifelong treatment with saline or BPAP

Age (months)		27	28	29	30	31	32	33	34	35	36	37	38	39	40	41	42	43
5CSRTT	Saline BPAP	15 15	15 15	14 15	13 15	11 13	10 13	10 13	8 12	8 10	7 8	6 6	6 5	6 2	5 1	4 1	1 1	1
COOP	Saline BPAP	14 14	14 14	14 14	12 14	10 12	10 12	8 12	8 11	8 9	7 7	5 5	6 5	6 1	5 1	4 1	1 1	
MWM	Saline BPAP	15 15	14 15	14 15	11 14	10 13	10 13	8 12	7 10	7 9	7 7	6 4						
PJ	Saline BPAP	15 15	12 15	12 15	11 15	10 12	10 12	8 12	8 10	7 8		6 5	4 1					

**Fig. 2:**
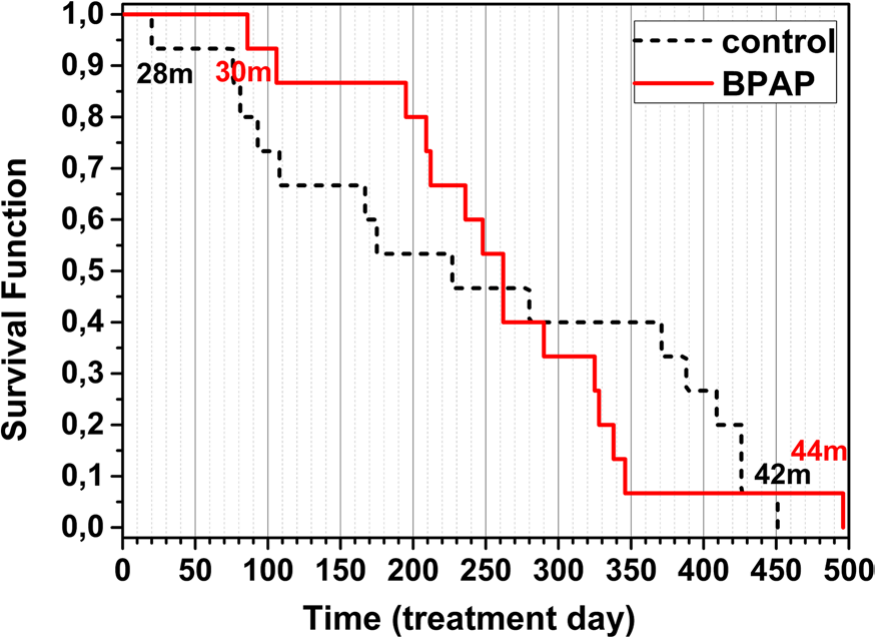
Survival analysis of rats participating in BPAP longevity study. The red solid curve indicate the BPAP-treated individuals, died on a given treatment day (marked on *x* axis), the black dashed curve indicate the control animals. Data show the age of the animals in months that died as first (control: 28 months, BPAP: 30 months) and last (control: 42 months, BPAP: 44 months) of the two treatment groups

During the treatment period, the survival of the rats was monitored. Until treatment day 260 (age of 36 months, *c.f.* Table 1), BPAP-treated rats died at a lower rate than the control-treated ones. Afterwards, BPAP-treated animals died at a higher rate than controls (Fig. 2). However, there was no significant difference between the survival curves of the two groups compared by Cox-Mantel test (*p* = 0.81). The last control rat died at age of 42 months, and the last rat of this longevity study was a BPAP-treated rat that died at age of 44 months. Our control animals lived in average 35.7 ± 1.3 months, while BPAP-treated animals’ lifespan was 36.2 ± 0.9 months.

In gross biopsy findings, there was no notable difference between the two groups: see *Supplementary material*, Table S5.

**Fig. 3:**
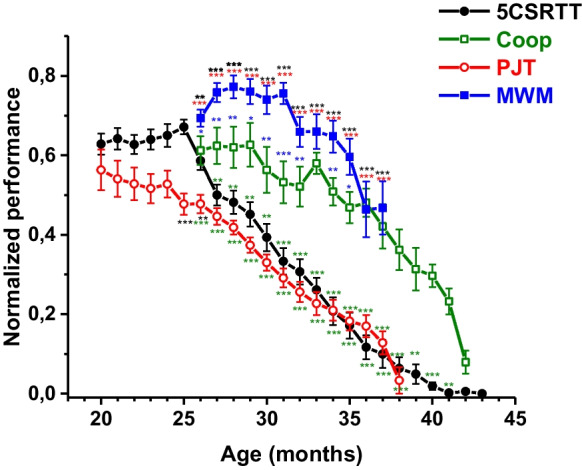
Normalized performance of rats depending on their age in four tasks: percent of successful trials in 5CSRTT (black curve, filled circle symbols), longest spanned distance in “pot jumping” (PJT) experiment (red curve, empty circle symbols), percent successful trials in cooperation task (Coop) (green curve, empty rectangle symbols), and “remaining time” in MWM task (blue curve, filled rectangle symbols). Data of BPAP-treated and control animals are pooled. The number of animals are listed in Table 1. Asterisks indicate the significant differences among monthly performance of the four tasks, analyzed by univariate ANOVA and Duncan test. Asterisks placed close to symbols of a given curve show the significance to the other curve having the same color as that of the asterisk

As in all the applied tasks, no significant difference was found between BPAP-treated and vehicle-treated groups; we pooled the data of the two groups when we compared the age-dependent changes in the rats’ performance among the four tasks (Fig. 3). According to the monthly data standardized by range, performance in pot jumping started to impair first (from the age of month 21). Afterwards (from month 26), the percent of successful trials in 5CSRTT task followed to degrade. Performance in Morris water maze navigation task became lower just from 32 months of age. Cooperation task that needed to be solved in pairs showed an impairing tendency in performance as last among the tasks, i.e., at 34 months of age.

## Discussion

### Comparing the performances of BPAP- and vehicle-treated groups

No significant difference was found in any of the four tasks with the only exception in MWM task at 33 months of age; however, no meaningful biological effect can be ascribed to this result. Not only in age-related cognitive abilities, but also in life extension BPAP did not seem to exert a considerable effect in the current study. The described positive effects of the compound [27, 43] could not be observed under the circumstances of the present study. There might be several explanations for this. (1) Strain difference: Knoll and Miklya [27] performed the longevity study in Wistar rats, in that BPAP-treated animals showed a better performance than the saline-treated rats using a shuttle box technique. Also, the lifespan of BPAP-treated rats was extended compared to the saline-treated rats. However, results with the pharmacological congener, deprenyl, in previous studies proved an increase of life span in rats of a different strain too [28–30] or even in other species [31–35]. (2) Starting time of treatment: According to Knoll and Miklya [27], BPAP showed an enhancer effect when it was administered from 2 months of age, whereas in our study, BPAP treatment started at much older age (27 months). However, studies with deprenyl demonstrated the life extension effect in rats after treatment starting at 2 years age [26, 28] or in a subset of elderly dogs with 10–15 years age range at initiation of administration [34]. (3) Dose of BPAP: The initial dose of BPAP (0.0002 mg /kg) did not produce an improvement in the performance of the rats during the first 7 weeks of the current study; thus, it was increased to 0.001 mg/kg for the rest of the study. It was tenfold higher than the dose of BPAP in the study of Knoll and Miklya [27]. This might have influenced the expected enhancer effect, although this dose of BPAP still falls into the “specific enhancer effect” range defined by Knoll and Miklya [27], and BPAP was equally active in prolonging life span at a higher dose, 0.05 mg/kg dose as well. (4) “Ceiling effect”: In the study of Knoll and Miklya [27], BPAP prolonged the average lifetime from days to 749 days, whereas in our study, even the control animals lived until 1071 days on average. It is already a very long lifespan, which may be difficult to prolong further (a more detailed elaboration of this point follows below). In accordance with this assumption, in most of the rat studies with deprenyl, the increased average lifespan remained below 950 days [28–30]. Nevertheless, in one study, deprenyl could increase lifespan from 1029 to 1343 days [26]. Overall, the enhancer and life extending effect of BPAP may be influenced by the interaction of all the above factors, i.e., strain of the rats, dose of the drug, age of the animals at initiation of the treatment, and their “baseline” lifespan.

### Life span of the animals

The longest living rat was a BPAP-treated rat that died at age of 44 months. According to a comparison between rat age and human age, 44 months of a rat age corresponds to about 110 human years [44]. Considering our whole tested rat population — independently of the treatment — they lived in average for 36.0 ± 0.8 months, which correspond to 90 human years [44]. The human male life expectancy at 68 years (which corresponds to 27 months rat age, when the treatment started) is 82 years (https://www.health.ny.gov/health_care/medicaid/publications/docs/adm/06adm-5att8.pdf). Using these statistical data, interestingly, our rats, “if they had been humans,” lived 8 years longer than the life expectancy calculated at the beginning of their treatment. We attribute this long life span to two major factors: food restriction and continuous cognitive activity.

We were keeping our animals under restricted food access during their full lifetime (see “Materials and methods” for details). Food restriction has repeatedly been shown to slow the aging process and the age-associated increase in mortality rate [45–48] as well as to prolong cognitive functioning [49–51]. This is in accordance with the here presented well-preserved physical and cognitive activity of our food-restricted rats. Regular and high level cognitive activity is well known to be a protective factor against age-related dementias in humans [52–54], and increased life expectancy of those with higher educational attainment is a statistical evidence [55-57]. In rodents, the effect of environmental enrichment (i.e., increased sensory stimulation) has typically been studied and shown to be beneficial on aged cognitive performance [58–63], but the positive effect of lifelong cognitive training was also demonstrated [64]. The regular daily activities of our rats in the various cognitive paradigms may have ensured — beside their direct brain stimulating effects — a highly effective environmental enrichment as well. Thus, owing to these “anti-aging” factors, cognitive improvement might have reached a ceiling, which may be one reason why the enhancer effect of BPAP could not be detected on lifespan and/or cognition in our animals.

### Age-related cognitive decline

According to our results, motor (pot jumping) performance started to impair first, at 21 months of age. Age-dependent decline in motor performance has been reported in numerous studies usually comparing a young and an old group. In studies which used a finer resolution (i.e. applied several age groups), impairment in motor performance compared to 4–6 months young rats was already shown from the age of 12–15 months, depending on the motor function measured [65–68]. In studies which examined motor performance in age groups above 20 months, a further impairment was observed in most of the motor functions [66–68]. Two longitudinal studies [68, 69] followed the animals up to the age of 30 months and reported a gradual decrease in motor functions, similarly to our findings. The decline observed in our study might be explained by age-related physical weakness and/or lack of motivation to jump through the already familiar environment, where the task was repeatedly done. We observed the appearance of “frailty” in our rat population from 27 months of age [20]; thus, lack of motivation might be the primary factor, especially as rats were freely allowed to move on the pots, that is, beside the exploratory drive nothing forced them to move.

In the performance in 5CSRTT, measuring attention started to impair through aging secondly (at 26 months of age). The decrease in percentage of successful trials was paralleled by an increase in the percentage of omitted responses, while response accuracy (percentage of correct responses in relation to correct + incorrect responses) remained relatively stable until the age of 33 months (see *Supplementary material*, Fig. S1) indicating again decreased motivation as the underlying factor of impairment in performance. Sustained attention requires high energy demand, and it may not have been worth for the rats to solve the task for a relatively low benefit: Reward pellets may not have been a sufficient motivating factor as aged rats’ food demand was getting lower. In two cross-sectional studies [59, 70], 2.5-year-old rats showed similar choice accuracy to that of 7 months and 1.5-year-old rats but with much higher omission rate. In contrast, a longitudinal study [71] of female Sprague–Dawley rats, being tested weekly, showed a reduction in choice accuracy (81–65%) between 12 and 23 months of age without changes in errors of omission making the authors exclude a role for motivational impairments. The latter result points out that female rats may age in a different way than males. It also limits the conclusions of the present study, which was only performed in male rats. Regretfully, this flaw is characteristic for the majority of the corresponding literature.

As third, navigation performance in Morris water maze started to decline. This task is aversively motivating: Rats need to strain themselves to escape from the water; hence, in this task, a lack of motivation may not cause a lower performance like in 5CSRTT or pot jumping tests. The major factor is physical weakness and the accompanying swimming difficulty which produces strong acute stress that may corrupt the navigational memory. MWM has become a very frequently used spatial learning assay [72, 73]; hence, its aging-related literature is enormous containing studies with the expected outcome of aged rats’ inferiority to young ones. However, investigation of aged rats’ behavior in this task does not spread as long as our rats were examined (37 months of age). Here, we only highlight a few studies with results relevant to our findings. The greater resistance of the Morris water maze task to aging than that of motor performance was also shown in a cross-sectional study of [65], who found that a working memory version of the Morris water maze task got impaired at 18 months’ age, 6 months later than complex motor learning. The constraining force of the aversive environment is demonstrated by the finding that Long Evans rats even older than 2 years were able to rapidly acquire this task [59, 74, 75]. However, it was also shown that 26-month-old naïve SPRD and 24-month-old naïve F-344 rats performed worse than their task-experienced conspecifics of the same age [13, 14].

Performance in cooperation task started to decline the latest, i.e., at 34 months of age. This task seems to be affected by age the least which we explain by a higher motivation of performing the task with a mate, in pairs. The mutual social support may primarily keep the motivation high and not the food reward. This kind of significance of social companion is supported by studies demonstrating that social interaction may provide a reward which suppresses drug self-administration and relapse to drug seeking [76, 77]. Lifelong cooperation performance was not examined yet in the literature; however, lifelong social housing was shown to prevent decline in working memory in rats [78].

In summary, we demonstrated that food restricted and cognitively engaged rats have a long lifespan and their cognitive performance in various tasks showed differential sensitivity/resistance to age related impairment. Our findings suggest that in this process, the primary factor is the level of motivation to perform and not losing the acquired knowledge. However, the enhancer substance, BPAP, could not slow down the course of aging and cognitive decline. Overall, learnt animals with “widespread knowledge” that we used in the present study provide a translationally relevant model to study age-related cognitive decline and measure the effect of putative anti-aging compounds.

### Supplementary Information

Below is the link to the electronic supplementary material.

Supplementary file1 (DOCX 104 KB)

## Data Availability

The data used in this study can be obtained on request from the authors.
