# Supplementary material for: Age-related decline of various cognitive functions in well-experienced male rats treated with the putative anti-aging compound (2R)-1-(1-benzofuran-2-yl)-N-propylpentane-2-amine ((-)BPAP)
Source: GeroScience. 2023 Jun 12;46(1):417–29. doi: 10.1007/s11357-023-00821-6 (PMC10828437; doi:10.1007/s11357-023-00821-6)
Supplement: Supplementary file 1 — Supplementary file1 (DOCX 104 KB) [file 11357_2023_821_MOESM1_ESM.docx]

**Supplementary materials**

Journal: **GeroScience**

Age-related decline of various cognitive functions in well-experienced male rats treated with the putative anti-aging compound (2R)-1-(1-benzofuran-2-yl)-N-propylpentane-2-amine ((-)BPAP)

Aliz Judit Ernyey^1^, Ferenc Kassai^1^, Kata Kozma^1^, Imola Plangár^1^, Zsuzsa Somfai^2^, Ildikó Miklya^2^, István Gyertyán^1^

^1^MTA-SE NAP B Cognitive Translational Behavioural Pharmacology Group,

Department of Pharmacology and Pharmacotherapy, Faculty of Medicine, Semmelweis University, Nagyvárad tér 4, H-1089, Budapest, Hungary

^2^Department of Pharmacology and Pharmacotherapy, Faculty of Medicine, Semmelweis University, Nagyvárad tér 4, H-1089, Budapest, Hungary

**Author note**

Corresponding author: Aliz Judit Ernyey

MTA-SE NAP B Cognitive Translational Behavioural Pharmacology Group,

Semmelweis University, Faculty of Medicine, Department of Pharmacology and Pharmacotherapy, Nagyvárad tér 4, H-1089 Budapest, Hungary

Email: ernyey.aliz@med.semmelweis-univ.hu

ORCID: 0000-0003-1957-2239

Table S1. Student’s t-tests for independent samples with Holm-Bonferroni correction in 5-choice serial reaction time task (5CSRTT)

| Month | Mean1 | Mean2 | t | df | p |
| --- | --- | --- | --- | --- | --- |
| Succ%27 | 40,33158 | 41,61660 | -0,249292 | 28 | 0,804955 |
| Succ%28 | 38,33411 | 39,05846 | -0,111904 | 28 | 0,911698 |
| Succ%29 | 32,20426 | 33,25213 | -0,182201 | 27 | 0,856786 |
| Succ%30 | 26,10830 | 25,42368 | 0,098225 | 26 | 0,922507 |
| Succ%31 | 25,64295 | 19,84478 | 1,148912 | 22 | 0,262931 |
| Succ%32 | 22,90190 | 24,48693 | -0,244205 | 21 | 0,809442 |
| Succ%33 | 15,04121 | 17,06966 | -0,298558 | 21 | 0,768212 |
| Succ%34 | 11,37412 | 9,15834 | 0,380582 | 18 | 0,707967 |
| Succ%35 | 11,96725 | 14,49760 | -0,383045 | 16 | 0,706728 |
| Succ%36 | 8,46336 | 2,27319 | 1,420378 | 13 | 0,179035 |
| Succ%37 | 6,63531 | 1,34046 | 0,867524 | 10 | 0,405992 |
| Succ%38 | 4,21354 | 5,03323 | -0,217002 | 9 | 0,833046 |
| Succ%39 | 0,00000 | 0,00000 |  | 6 |  |
| Succ%40 | 0,50000 | 0,00000 | 0,408248 | 4 | 0,704000 |
| Succ%41 | 0,00000 | 0,00000 |  | 3 |  |
| Succ%42 | 0,00000 | 0,00000 |  | 0 |  |
| Succ%43 |  | 0,00000 |  | 0 |  |

Table S2. Student’s t-tests for independent samples with Holm-Bonferroni correction in cooperation task (COOP)

| Month | Mean1 | Mean2 | t | df | p | Holm-Bonferroni –  corrected p | |
| --- | --- | --- | --- | --- | --- | --- | --- |
| Succ%31 | 30,61101 | 49,45353 | -2,63364 | 20 | 0,015923 | 0,23884 |  |
| Succ%34 | 40,57641 | 27,19776 | 1,94573 | 17 | 0,068413 | 0,957781 |  |
| Succ%38 | 30,44240 | 15,04621 | 1,87679 | 9 | 0,093283 | 1,212685 |  |
| Succ%36 | 38,91537 | 32,64628 | 1,23199 | 12 | 0,241549 | 2,898588 |  |
| Succ%37 | 30,39877 | 16,52609 | 1,24752 | 8 | 0,247487 | 2,898588 |  |
| Succ%28 | 43,09226 | 53,88693 | -1,16503 | 26 | 0,254585 | 2,545847 |  |
| Succ%32 | 41,11033 | 50,10552 | -1,10599 | 20 | 0,281856 | 2,5367 |  |
| Succ%39 | 24,82523 | 15,04855 | 1,20019 | 5 | 0,283823 | 2,270583 |  |
| Succ%30 | 27,44287 | 37,52677 | -1,06047 | 24 | 0,299485 | 2,096398 |  |
| Succ%35 | 38,81446 | 32,33024 | 0,89016 | 15 | 0,387433 | 2,324595 |  |
| Succ%41 | 2,75305 | 0,00000 | 0,77193 | 3 | 0,496388 | 2,48194 |  |
| Succ%29 | 40,00326 | 46,16836 | -0,67934 | 26 | 0,502925 | 2,0117 |  |
| Succ%40 | 20,21127 | 17,72336 | 0,70046 | 4 | 0,522244 | 1,566732 |  |
| Succ%33 | 37,87156 | 35,66675 | 0,33719 | 18 | 0,739877 | 1,479754 |  |
| Succ%27 | 52,09612 | 54,01570 | -0,21234 | 26 | 0,833494 | 0,833494 |  |
| Succ%42 | 0,00000 | 0,00000 |  | 0 |  | 0 |  |

Table S3. Student’s t-tests for independent samples with Holm-Bonferroni correction in Morris water-maze experiment (MWM)

| Month | Mean1 | Mean2 | t | df | p | Holm-Bonferroni –  corrected p |
| --- | --- | --- | --- | --- | --- | --- |
| Remaining33 | 70,76438 | 51,24750 | 3,33800 | 18 | 0,003661 | 0,0402682 |
| Remaining30 | 54,11364 | 65,49286 | -2,28993 | 23 | 0,031529 | 0,31529269 |
| Remaining35 | 55,65357 | 38,41722 | 1,79750 | 14 | 0,093852 | 0,84466834 |
| Remaining29 | 61,36155 | 70,11833 | -1,67646 | 27 | 0,105192 | 0,84466834 |
| Remaining36 | 48,73143 | 30,68000 | 1,62395 | 12 | 0,130346 | 0,91241973 |
| Remaining37 | 29,46000 | 53,09500 | -1,42109 | 8 | 0,193074 |  |
| Remaining31 | 69,37400 | 61,05692 | 0,90492 | 21 | 0,375773 |  |
| Remaining27 | 65,68711 | 69,06911 | -0,84757 | 28 | 0,403862 |  |
| Remaining34 | 59,90429 | 54,49850 | 0,66008 | 15 | 0,519209 |  |
| Remaining32 | 58,27400 | 55,02115 | 0,42555 | 21 | 0,674764 |  |
| Remaining28 | 67,64429 | 66,22800 | 0,25581 | 27 | 0,800037 |  |

Table S4. Student’s t-tests for independent samples with Holm-Bonferroni correction in „pot jumping” test (PJT)

| Month | Mean1 | Mean2 | t | df | p |
| --- | --- | --- | --- | --- | --- |
| LongestD27 | 25,33333 | 25,60000 | -0,409776 | 28 | 0,685089 |
| LongestD28 | 25,00000 | 25,20000 | -0,227862 | 25 | 0,821607 |
| LongestD29 | 23,75000 | 23,06667 | 0,662744 | 25 | 0,513559 |
| LongestD30 | 23,36364 | 23,66667 | -0,353568 | 24 | 0,726750 |
| LongestD31 | 21,70000 | 23,00000 | -0,995324 | 20 | 0,331469 |
| LongestD32 | 21,90000 | 21,50000 | 0,334604 | 20 | 0,741407 |
| LongestD33 | 22,50000 | 21,75000 | 0,742097 | 18 | 0,467602 |
| LongestD34 | 21,75000 | 20,10000 | 1,278724 | 16 | 0,219231 |
| LongestD35 | 20,78571 | 21,37500 | -0,559681 | 13 | 0,585212 |
| LongestD36 | 20,00000 | 18,00000 | 1,430194 | 9 | 0,186447 |
| LongestD37 | 18,00000 | 18,00000 |  | 3 |  |

Table S5. Autopsy results of BPAP- and vehicle-treated rats

|  | BPAP (N=12) | vehicle (N=13) |
| --- | --- | --- |
| tumor /fibroma | 6 | 5 |
| gastric ulcer, (presumed) | 6 | 5 |
| organ enlargement | 7 | 6 |
| liquid in chest or abdominal cavity | 1 | 5 |

Fig. S1. Percent of successful trials, accuracy and omission in 5-choice serial reaction time task. Data of BPAP-treated and control animals are pooled. Mean ± s.e.m values are shown. Regression lines fitted on the curves are shown in gray. Significant difference was found between the slopes of regression lines of percent successful trials and accuracy (p= 0.041, t-test for independent samples) but not between percent successful trials and the inverse of percent omissions (p=0.086).
